# Book review

**DOI:** 10.1155/S1463924697000205

**Published:** 1997

**Authors:** Paul Worsfold

**Affiliations:** Plymouth Environmental Research Centre University of Plymouth Drake Circus Plymouth PL4 8AA UK


### Book review

*Automatic Chemical Analysis*, by P. B. Stockwell (in association with W. T. Corns), Taylor & Francis, London (1996). ISBN 0-7484-0480-5, vi+239 pp. Price £55.00

This is a completely revised version of the popular first edition of *Automatic Chemical Analysis* published in 1974 and written by Peter Stockwell and the late Jim Foreman. Given the advances that have taken place in laboratory automation in recent years this is a timely contribution. What differentiates it from other recent monographs in this area is the practical nature of the contents, including an appraisal of the economic aspects of automation. This is particularly well emphasized by chapter 3, which presents three case studies in depth. These relate to the regulation of tar and nicotine in cigarettes, mercury analysis in a natural gas plant and water laboratory automation.

The first two chapters present an introduction to, and outline the principles of, automatic chemical analysis. The material will be familiar to readers of the first edition and includes discussions for (and against) automation, the philosophy of automation and the operation of various wet chemical analysers, for example, discrete, segmented continuous-flow and flow-injection based, the latter being a major development since the publication of the first edition.

One of the often neglected areas of laboratory automation is sample preparation and this is considered in detail in chapter 4, with particular focus on sample clean-up and sample digestion. The remaining chapters of the book cover automated atomic spectroscopy, robots and computers, examples of automatic systems (automatic specific gravity and refractive index measurement, computer-assisted analysis of metals, automated tablet dissolution, low level mercury analysis and automated pH and conductivity measurement) and a look into the future of automatic analysis. This last chapter focuses particularly on the increasing influence of computing power and the issue of system validation.

The book is clearly written and presented with a good number of figures and tables, a substantial index and a glossary of manufacturers' addresses. It is a worthy and timely successor to the 1974 edition, and its practical approach to the subject makes it recommended reading for laboratory managers and all students of analytical chemistry.

**Paul Worsfold**
*Plymouth Environmental Research Centre,*
*University of Plymouth, Drake Circus,*
*Plymouth PL4 8AA, UK*

0142-0453/97 $12·00 © 1997 Taylor & Francis Ltd

